# Nicotinamide drives T cell activation in the mammary tumor microenvironment

**DOI:** 10.1186/s12967-022-03454-z

**Published:** 2022-06-03

**Authors:** Yang Hu, Norma Bloy, Olivier Elemento, Aitziber Buqué

**Affiliations:** 1 Department of Physiology and Biophysics, Weill Cornell Medical College, New York, NY USA; 2 Department of Radiation Oncology, Weill Cornell Medical College, New York, NY USA; 3 Caryl and Israel Englander Institute for Precision Medicine, Weill Cornell Medical College, New York, NY USA

**Keywords:** CTLA4, Immune checkpoint inhibitors, Immunotherapy, PD-1, LAG3, TIM-3

## Abstract

Nicotinamide (NAM, a variant of vitamin B_3_) has recently been shown to accelerate the activation of human CD4^+^ and CD8^+^ T cells exposed to repeated CD3/CD28 agonism in vitro. Here, we demonstrate that T cells infiltrating mouse mammary carcinomas that are therapeutically controlled by NAM also express multiple markers of late-stage activation. Taken together, these findings lend additional support to the notion that the antineoplastic effects of NAM involve at least some degree of restored cancer immunosurveillance.

**Supplementary Information:**

The online version contains supplementary material available at 10.1186/s12967-022-03454-z.

Dear Editor,

Nicotinamide (NAM) is a variant of vitamin B_3_ that has been shown to mediate antineoplastic effects in a variety of tumor models [1], but the underlying mechanisms remain to be completely understood.

Recent findings from Alavi and colleagues demonstrate that NAM accelerates the acquisition of a polyfunctional cytokine expression profile, which involves the co-expression of interleukin 2 (IL2), interferon gamma (IFNG) and tumor necrosis factor (TNF), by human CD8^+^ T cells repeatedly exposed to CD3/CD28 agonism in vitro. Alongside, NAM promoted T cell differentiation towards a terminally differentiated effector memory (T_EMRA_) phenotype as it prevented the acquisition of exhaustion markers including hepatitis A virus cellular receptor 2 (HAVCR2, a co-inhibitory receptor best known as TIM-3) and ectonucleoside triphosphate diphosphohydrolase 1 (ENTPD1, an extracellular enzyme that initiates the conversion of immunostimulatory ATP into immunosuppressive adenosine), at least in the CD8^+^ T cell compartment [2]. All these events were paralleled by the inhibited upregulation of the transcription factor thymocyte selection associated high mobility group box (TOX), which is intimately involved in T cell exhaustion [3], and the epigenetic regulator enhancer of zeste 2 polycomb repressive complex 2 subunit (EZH2), which negatively controls TOX levels, even though no effects were noted on EZH2-dependent histone 3 K27 trimethylation [2].

In 2020, we reported the ability of NAM to mediate prophylactic and therapeutic effects in several mouse models of hormone receptor (HR)-positive and triple-negative breast cancer, including not only TS/A and AT3 cells established in immunocompetent syngeneic mice, but also endogenous mammary carcinomas driven in immunocompetent C57BL/6 mice by subcutaneous slow-release medroxyprogesterone acetate (MPA) pellets combined with oral 7,12-dimethylbenz (a) anthracene [1]. Importantly, both the prophylactic and the therapeutic activity of NAM could be limited by a variety of interventions that decrease the immunological competence of the host, including the co-depletion of CD4^+^ and CD8^+^ T cells as well as the neutralization of IFNG [1]. Moreover, the immune infiltrate of NAM-treated tumors exhibited multiple genetic and phenotypic signs of accrued immunological competence [1].

Inspired by the findings from Alavi and colleagues [2], we re-interrogated single cell RNA sequencing (scRNAseq) data obtained from the lymphoid compartment of TS/A mouse mammary carcinomas established in immunocompetent syngeneic mice that were either left untreated or received NAM supplementation with the drinking water, as per the experimental procedures reported in Ref. [1]. Since the original sequencing data does not allow for a clear distinction between the CD4^+^ and CD8^+^ T cell compartment in the context of preserved statistical power, we decided to focus on CD3^+^ T cells as a whole.

T cells from NAM-treated TS/A tumors exhibited a significant increase in the levels of multiple genes involved in TCR signaling (*Cd3d*, *Cd3e*, *Cd3g*, *Cd8a, Cd8b1*, *Lck*), CD8^+^ T cell effector functions including cytotoxicity (*Fasl, Gzmb, Nkg7*) and CD4^+^ helper T cell immunostimulation (*Mif*), bioenergetic metabolism (*Atp5j*, *Cox6a1*, *Ldha*, *Ndufa8*, *Ndufa13*, *Ndufaf4*, *Ndufb2*, *Ndufs8*), IFNG signaling (*Ifi47*) as well as genes encoding late-stage activation/exhaustion markers (*Klrc1*, *Klrc2*, *Klrd1*, *Pdcd1*). Conversely, NAM supplementation with the drinking water was associated to the downregulation of genes encoding inhibitors of cell cycle progression (*Cdk2ap1*, *Cdkn1b*), chemotactic factors (*Ccl8*, *Cxcl1*), and positive regulators of quiescence (*Btg1*), as well as multiple genes preferentially expressed by immunosuppressive regulatory T (T_REG_) cells (*Edr1*, *Gnai2*, *Klf2*, *Lgmn*) (Fig. 1 and Additional file 1: Table S1).

**Fig. 1:**
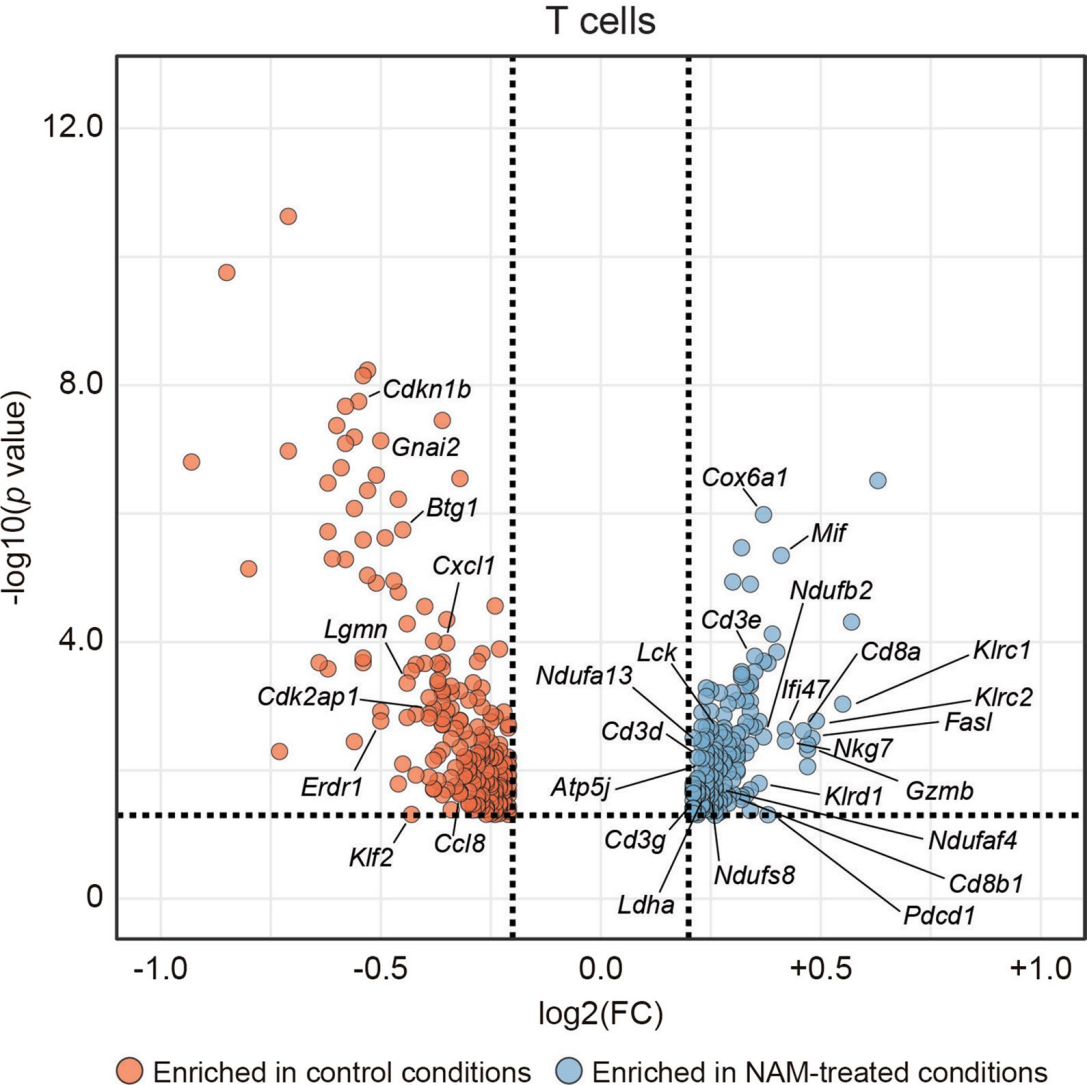
NAM-driven differential gene expression in TS/A-infiltrating lymphoid cells. Six-to-eight weeks-old wild-type female BALB/c mice were implanted with 0.1 × 10^6^ mouse mammary adenocarcinoma TS/A cells *s.c.* and left untreated until tumor surface reached 20–40 mm^2^. Thereafter, TS/A-bearing mice were randomized to standard drinking water or drinking water supplemented with 0.5% (w/v) NAM for ten days, when tumors were collected and processed for scRNAseq as detailed in Ref. [1]. Volcano plots on differentially expressed genes are reported for CD45^+^CD3 ^+^ T cells. Genes of interest are labelled. Thresholds for absolute log2 fold change (FC) and adjusted *p* value have been arbitrarily set to > 0.2 and < 0.05, respectively. scRNAseq data were analyzed as detailed in Ref. [1]. See also Additional file 1: Table S1

Together with the in vitro data discussed above [2] as well as in vivo data from preclinical models of pancreatic cancer [4], our findings corroborate the notion that the antineoplastic effects of NAM involve a considerable immunological component. While dosing considerations may prevent NAM from being employed as a direct approach to treat cancer, a clinical trial investigating NAM as a means to improve the ex vivo expansion of natural killer (NK) cells for haploidentical or mismatched related transplantation in patients with hematological malignancies is currently recruiting participants (NCT03019666). If successful, this study may set the foundations to the use of NAM in ex vivo T and NK cell expansion procedures, which has considerable implications not only for hematological transplants but also for novel cell-based immunotherapies including CAR-expressing T cells [5].

## Supplementary Information

**Additional file 1.** Genes of interest differentially expressed in T cells from NAM-treated vs control TS/A tumors.

## Data Availability

The datasets analyzed during the current study are available from the corresponding author on reasonable request.
