# Supplementary material for: Nicotinamide drives T cell activation in the mammary tumor microenvironment
Source: J Transl Med. 2022 Jun 3;20:251. doi: 10.1186/s12967-022-03454-z (PMC9164530; doi:10.1186/s12967-022-03454-z)
Supplement: Supplementary file 1 — Additional file 1. Genes of interest differentially expressed in T cells from NAM-treated vs control TS/A tumors. [file 12967_2022_3454_MOESM1_ESM.pdf]

Supplemental Table 1. Genes of interest differentially expressed in T cells from NAM-treated *vs* control TS/A tumors.

Gene	log2(FC)	*p* value	- log10(*p* value)
Klrc1	0.55	9.26E-04	3.03
Klrc2	0.49	1.70E-03	2.77
Fasl	0.48	3.17E-03	2.5
Gzmb	0.47	4.78E-03	2.32
Cd8a	0.46	2.42E-03	2.62
Ifi47	0.42	2.32E-03	2.63
Nkg7	0.42	3.50E-03	2.46
Mif	0.41	4.49E-06	5.35
Pdcd1	0.38	4.90E-02	1.31
Ndufb2	0.37	3.01E-03	2.52
Cox6a1	0.37	1.04E-06	5.98
Klrd1	0.36	1.59E-02	1.8
Cd3e	0.35	1.67E-04	3.78
Cd8b1	0.29	2.50E-02	1.6
Lck	0.27	2.60E-03	2.59
Ndufaf4	0.26	1.85E-02	1.73
Ndufs8	0.25	2.26E-02	1.65
Ldha	0.24	2.12E-02	1.67
Atp5j	0.24	7.87E-03	2.1
Ndufa13	0.23	4.00E-03	2.4
Cd3d	0.23	6.46E-03	2.19
Cd3g	0.20	2.80E-02	1.55
Ccl8	-0.32	2.21E-02	1.66
Cxcl1	-0.35	1.04E-04	3.98
Cdk2ap1	-0.38	1.10E-03	2.96
Klf2	-0.43	4.86E-02	1.31
Lgmn	-0.44	4.37E-04	3.36
Btg1	-0.45	1.79E-06	5.75
Gnai2	-0.50	7.34E-08	7.13
Erdr1	-0.50	1.70E-03	2.77
Cdkn1b	-0.55	1.79E-08	7.75

Abbreviations. FC, fold change; n/a, not applicable; NAM, nicotinamide.
